# Diethyl 6,9,17,20-tetra­bromo-2,13-di­oxo­hexa­cyclo­[10.10.2.0^3,24^.0^5,10^.0^14,23^.0^16,21^]tetra­cosa-5,7,9,16,18,20-hexa­ene-23,24-dicarboxyl­ate

**DOI:** 10.1107/S1600536809009490

**Published:** 2009-03-19

**Authors:** Yanping Zhu, Yan Chen, Yichong Sun

**Affiliations:** a Key Laboratory of Pesticides and Chemical Biology of the Ministry of Education, College of Chemistry, Central China Normal University, Wuhan 430079, People’s Republic of China

## Abstract

In the title mol­ecule, C_26_H_22_Br_4_N_4_O_6_, the dihedral angle between the aromatic rings is 30.0 (1)°. One ethyl fragment is disordered between two positions in a 1:1 ratio. The crystal packing exhibits weak inter­molecular C—H⋯O hydrogen bonds and short Br⋯O contacts of 3.349 (6) Å.

## Related literature

For applications of glycoluril derivatives, see: Wu *et al.* (2002 ▶); Lee *et al.* (2003 ▶); Rowan *et al.* (1999 ▶); Hof *et al.* (2002 ▶). For details of the synthesis, see: Chen *et al.* (2007 ▶).
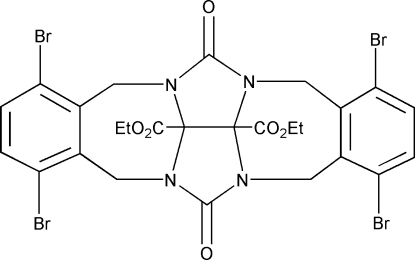


## Experimental

### 

#### Crystal data

C_26_H_22_Br_4_N_4_O_6_
*M* *_r_* = 806.12
Monoclinic, 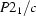

*a* = 12.3545 (16) Å
*b* = 16.256 (2) Å
*c* = 13.8793 (18) Å
β = 93.396 (2)°
*V* = 2782.5 (6) Å^3^
*Z* = 4
Mo *K*α radiation
μ = 5.83 mm^−1^
*T* = 292 K
0.30 × 0.20 × 0.20 mm

#### Data collection

Bruker SMART 4K CCD area-detector diffractometer
Absorption correction: none
18842 measured reflections
6319 independent reflections
3100 reflections with *I* > 2σ(*I*)
*R*_int_ = 0.056

#### Refinement

*R*[*F* ^2^ > 2σ(*F* ^2^)] = 0.054
*wR*(*F* ^2^) = 0.136
*S* = 1.00
6319 reflections
371 parameters
4 restraints
H-atom parameters constrained
Δρ_max_ = 0.56 e Å^−3^
Δρ_min_ = −0.45 e Å^−3^

### 

Data collection: *SMART* (Bruker, 1997 ▶); cell refinement: *SAINT* (Bruker, 1999 ▶); data reduction: *SAINT*; program(s) used to solve structure: *SHELXS97* (Sheldrick, 2008 ▶); program(s) used to refine structure: *SHELXL97* (Sheldrick, 2008 ▶); molecular graphics: *PLATON* (Spek, 2009 ▶); software used to prepare material for publication: *SHELXL97* and *PLATON*.

## Supplementary Material

Crystal structure: contains datablocks I, global. DOI: 10.1107/S1600536809009490/cv2524sup1.cif

Structure factors: contains datablocks I. DOI: 10.1107/S1600536809009490/cv2524Isup2.hkl

Additional supplementary materials:  crystallographic information; 3D view; checkCIF report

## Figures and Tables

**Table 1:** Hydrogen-bond geometry (Å, °)

*D*—H⋯*A*	*D*—H	H⋯*A*	*D*⋯*A*	*D*—H⋯*A*
C4—H4⋯O2^ii^	0.93	2.36	3.278 (7)	172
C19—H19*A*⋯O3^iii^	0.97	2.27	3.227 (7)	169
C25—H25⋯O2^iv^	0.93	2.56	3.309 (7)	138

Symmetry codes: (ii) 
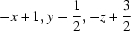
; (iii) 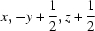; (iv) 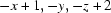.

**Fig. 1.**
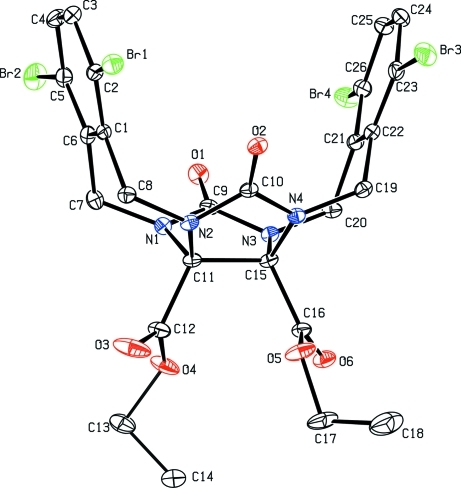
View of the molecule of (I) showing the atom-labelling scheme. Displacement ellipsoids are drawn at the 30% probability level. H atoms are represented by spheres of arbitrary radius. Only major parts of disordered atoms are shown.

## supplementary crystallographic information

### Comment

Glycoluril derivatives have manifested applications in many fields, such as explosives, slow-release fertilizers, crosslinkers, stabilizers of organic compounds agaist photodegratation and reagents in combinational chemistry (Wu *et al.*, 2002). They also play an important role in building block for the preparation of a wide variety of supramolecular assemble, including cucurbit[*n*] uril homologues (n = 5, 7, 8 and 10) and their derivatives (Lee *et al.*, 2003), molecular clips and baskets (Rowan *et al.*, 1999), and molecular capsules (Hof *et al.*, 2002).

Herein, we present the X-ray crystal structure of the title compound, which is derivated from diethoxycarbonyl glycoluril with dibromo-substituted benzene ring fusing to the side-wall of the molecular clip. The distance between the two carbonyl oxygen atoms (O1—O2) of the glycoluril fragment is 5.498 (7) Å. The crystal packing exhibits weak intermolecular C—H···O hydrogen bonds (Table 2) and short Br···O contacts of 3.349 (6) Å (Table 1).

### Experimental

The title compound was synthesized acoording to the procedure reported by Chen *et al.* (2007). Crystals appropriate for data collection were obtained by slow evaporation of a methanol-chloroform (1:30 *v*/*v*) solution at 283 K.

### Refinement

All H atoms were initially located in a difference Fourier map and then included in the refinement with constrained bond lengths and isotropic displacement parameters: C—H = 0.93 Å and U_iso_(H) = 1.2 U_eq_(C) for aromatic H atoms, C—H = 0.97 Å and U_iso_(H) = 1.2 U_eq_(C) for methylene H atoms, C—H = 0.96 Å and U_iso_(H) = 1.5 U_eq_(C) for methyl H atoms.

### Crystal data

**Table:**

C_26_H_22_Br_4_N_4_O_6_	*F*(000) = 1576
*M**_r_* = 806.12	*D*_x_ = 1.924 Mg m^−^^3^
Monoclinic, *P*2_1_/*c*	Mo *K*α radiation, λ = 0.71073 Å
Hall symbol: -p/2ybc	Cell parameters from 2976 reflections
*a* = 12.3545 (16) Å	θ = 2.6–20.7°
*b* = 16.256 (2) Å	µ = 5.83 mm^−^^1^
*c* = 13.8793 (18) Å	*T* = 292 K
β = 93.396 (2)°	Block, colourless
*V* = 2782.5 (6) Å^3^	0.30 × 0.20 × 0.20 mm
*Z* = 4	

### Data collection

**Table:**

Bruker SMART 4K CCD area-detector diffractometer	3100 reflections with *I* > 2σ(*I*)
Radiation source: fine-focus sealed tube	*R*_int_ = 0.056
graphite	θ_max_ = 27.5°, θ_min_ = 1.7°
φ and ω scans	*h* = −12→15
18842 measured reflections	*k* = −21→21
6319 independent reflections	*l* = −17→17

### Refinement

**Table:**

Refinement on *F*^2^	Primary atom site location: structure-invariant direct methods
Least-squares matrix: full	Secondary atom site location: difference Fourier map
*R*[*F*^2^ > 2σ(*F*^2^)] = 0.054	Hydrogen site location: inferred from neighbouring sites
*wR*(*F*^2^) = 0.136	H-atom parameters constrained
*S* = 1.00	*w* = 1/[σ^2^(*F*_o_^2^) + (0.0515*P*)^2^ + 0.6576*P*] where *P* = (*F*_o_^2^ + 2*F*_c_^2^)/3
6319 reflections	(Δ/σ)_max_ = 0.002
371 parameters	Δρ_max_ = 0.56 e Å^−^^3^
4 restraints	Δρ_min_ = −0.45 e Å^−^^3^

### Special details

**Table:**

Geometry. All e.s.d.'s (except the e.s.d. in the dihedral angle between two l.s. planes) are estimated using the full covariance matrix. The cell e.s.d.'s are taken into account individually in the estimation of e.s.d.'s in distances, angles and torsion angles; correlations between e.s.d.'s in cell parameters are only used when they are defined by crystal symmetry. An approximate (isotropic) treatment of cell e.s.d.'s is used for estimating e.s.d.'s involving l.s. planes.
Refinement. Refinement of *F*^2^ against ALL reflections. The weighted *R*-factor *wR* and goodness of fit *S* are based on *F*^2^, conventional *R*-factors *R* are based on *F*, with *F* set to zero for negative *F*^2^. The threshold expression of *F*^2^ > σ(*F*^2^) is used only for calculating *R*-factors(gt) *etc*. and is not relevant to the choice of reflections for refinement. *R*-factors based on *F*^2^ are statistically about twice as large as those based on *F*, and *R*- factors based on ALL data will be even larger.

### Fractional atomic coordinates and isotropic or equivalent isotropic displacement parameters (Å^2^)

**Table:**

	*x*	*y*	*z*	*U*_iso_*/*U*_eq_	Occ. (<1)
Br1	0.61969 (5)	0.07496 (4)	0.61428 (4)	0.0694 (2)	
Br2	0.25324 (7)	−0.20733 (4)	0.66394 (5)	0.0933 (3)	
Br3	0.55658 (5)	0.17500 (5)	1.06195 (5)	0.0857 (3)	
Br4	0.15555 (6)	−0.08284 (4)	1.09507 (5)	0.0769 (2)	
C1	0.3996 (4)	0.0218 (3)	0.6329 (3)	0.0433 (13)	
C2	0.5079 (4)	−0.0014 (4)	0.6327 (3)	0.0508 (14)	
C3	0.5384 (5)	−0.0833 (4)	0.6472 (4)	0.0653 (17)	
H3	0.6113	−0.0979	0.6500	0.078*	
C4	0.4613 (6)	−0.1412 (4)	0.6572 (4)	0.0678 (17)	
H4	0.4813	−0.1961	0.6648	0.081*	
C5	0.3539 (5)	−0.1196 (4)	0.6564 (4)	0.0588 (16)	
C6	0.3195 (4)	−0.0390 (3)	0.6449 (3)	0.0462 (13)	
C7	0.2015 (4)	−0.0159 (3)	0.6420 (4)	0.0526 (14)	
H7A	0.1814	0.0081	0.5795	0.063*	
H7B	0.1588	−0.0655	0.6482	0.063*	
C8	0.3652 (4)	0.1101 (3)	0.6156 (3)	0.0469 (13)	
H8A	0.4291	0.1427	0.6047	0.056*	
H8B	0.3180	0.1125	0.5573	0.056*	
C9	0.1494 (4)	0.0176 (4)	0.8077 (4)	0.0449 (13)	
C10	0.3658 (4)	0.1654 (3)	0.7802 (4)	0.0436 (12)	
C11	0.1973 (4)	0.1283 (3)	0.7108 (3)	0.0427 (12)	
C12	0.1199 (5)	0.1683 (4)	0.6322 (4)	0.0649 (17)	
C13	−0.0687 (11)	0.1651 (10)	0.5741 (10)	0.080 (4)	0.541 (11)
H13A	−0.1120	0.1154	0.5676	0.096*	0.541 (11)
H13B	−0.0414	0.1791	0.5121	0.096*	0.541 (11)
C14	−0.1315 (18)	0.2345 (11)	0.6141 (16)	0.107 (6)	0.541 (11)
H14A	−0.1431	0.2240	0.6808	0.160*	0.541 (11)
H14B	−0.2003	0.2393	0.5785	0.160*	0.541 (11)
H14C	−0.0916	0.2848	0.6087	0.160*	0.541 (11)
C15	0.1837 (4)	0.1587 (3)	0.8168 (3)	0.0441 (13)	
C16	0.1066 (5)	0.2326 (4)	0.8243 (4)	0.0523 (14)	
C17	0.0825 (7)	0.3716 (5)	0.7716 (7)	0.109 (3)	
H17A	0.0886	0.3939	0.7073	0.131*	
H17B	0.0071	0.3575	0.7785	0.131*	
C18	0.1129 (7)	0.4310 (5)	0.8384 (8)	0.155 (4)	
H18A	0.0864	0.4165	0.8998	0.232*	
H18B	0.0829	0.4830	0.8179	0.232*	
H18C	0.1905	0.4348	0.8441	0.232*	
C19	0.3251 (4)	0.1945 (3)	0.9489 (4)	0.0485 (13)	
H19A	0.2710	0.2292	0.9764	0.058*	
H19B	0.3929	0.2248	0.9519	0.058*	
C20	0.1437 (4)	0.0819 (3)	0.9678 (3)	0.0507 (14)	
H20A	0.0931	0.0395	0.9850	0.061*	
H20B	0.1180	0.1336	0.9926	0.061*	
C21	0.2533 (4)	0.0630 (3)	1.0178 (3)	0.0474 (13)	
C22	0.3400 (4)	0.1176 (3)	1.0107 (3)	0.0463 (13)	
C23	0.4376 (4)	0.1014 (4)	1.0606 (4)	0.0548 (15)	
C24	0.4532 (5)	0.0293 (4)	1.1130 (4)	0.0637 (17)	
H24	0.5205	0.0181	1.1438	0.076*	
C25	0.3699 (5)	−0.0252 (4)	1.1194 (4)	0.0612 (16)	
H25	0.3799	−0.0738	1.1543	0.073*	
C26	0.2702 (4)	−0.0073 (4)	1.0733 (4)	0.0541 (14)	
C13'	−0.0469 (11)	0.2140 (12)	0.5903 (17)	0.080 (4)	0.459 (11)
H13C	−0.0312	0.2114	0.5227	0.096*	0.459 (11)
H13D	−0.0380	0.2701	0.6133	0.096*	0.459 (11)
C14'	−0.1574 (15)	0.1810 (14)	0.607 (2)	0.107 (6)	0.459 (11)
H14D	−0.1635	0.1257	0.5830	0.160*	0.459 (11)
H14E	−0.2114	0.2149	0.5743	0.160*	0.459 (11)
H14F	−0.1681	0.1811	0.6751	0.160*	0.459 (11)
N1	0.1740 (3)	0.0420 (3)	0.7170 (3)	0.0441 (10)	
N2	0.3088 (3)	0.1470 (2)	0.6948 (3)	0.0433 (10)	
N3	0.1414 (3)	0.0873 (3)	0.8634 (3)	0.0431 (10)	
N4	0.2926 (3)	0.1793 (2)	0.8489 (3)	0.0432 (10)	
O1	0.1336 (3)	−0.0520 (2)	0.8333 (3)	0.0616 (10)	
O2	0.4638 (3)	0.1696 (2)	0.7916 (3)	0.0531 (9)	
O3	0.1497 (4)	0.2044 (4)	0.5651 (4)	0.130 (2)	
O4	0.0196 (3)	0.1562 (3)	0.6488 (3)	0.0963 (17)	
O5	0.1485 (4)	0.2968 (3)	0.7819 (4)	0.0913 (15)	
O6	0.0233 (3)	0.2313 (3)	0.8621 (3)	0.0691 (12)	

### Atomic displacement parameters (Å^2^)

**Table:**

	*U*^11^	*U*^22^	*U*^33^	*U*^12^	*U*^13^	*U*^23^
Br1	0.0487 (4)	0.0843 (5)	0.0763 (4)	0.0001 (3)	0.0146 (3)	−0.0019 (3)
Br2	0.1236 (7)	0.0512 (5)	0.1055 (6)	−0.0248 (4)	0.0106 (5)	−0.0040 (4)
Br3	0.0615 (5)	0.1006 (6)	0.0927 (5)	−0.0176 (4)	−0.0144 (4)	−0.0126 (4)
Br4	0.0786 (5)	0.0652 (5)	0.0865 (5)	−0.0051 (4)	0.0010 (4)	0.0304 (4)
C1	0.052 (3)	0.042 (3)	0.035 (3)	0.006 (3)	0.001 (2)	0.000 (2)
C2	0.049 (4)	0.059 (4)	0.045 (3)	0.009 (3)	0.007 (2)	−0.002 (3)
C3	0.069 (4)	0.068 (5)	0.059 (3)	0.020 (4)	0.002 (3)	−0.010 (3)
C4	0.083 (5)	0.048 (4)	0.073 (4)	0.017 (4)	0.008 (4)	−0.003 (3)
C5	0.075 (5)	0.052 (4)	0.049 (3)	−0.004 (3)	0.003 (3)	−0.003 (3)
C6	0.058 (4)	0.041 (4)	0.040 (3)	−0.004 (3)	0.006 (2)	−0.002 (2)
C7	0.050 (4)	0.057 (4)	0.051 (3)	−0.015 (3)	−0.001 (3)	−0.002 (3)
C8	0.040 (3)	0.052 (4)	0.049 (3)	0.001 (3)	0.011 (2)	0.007 (3)
C9	0.031 (3)	0.045 (4)	0.057 (3)	−0.007 (3)	−0.004 (2)	0.008 (3)
C10	0.043 (4)	0.027 (3)	0.060 (3)	−0.002 (2)	0.001 (3)	0.005 (2)
C11	0.034 (3)	0.040 (3)	0.054 (3)	0.002 (2)	0.005 (2)	0.009 (2)
C12	0.050 (4)	0.087 (5)	0.058 (4)	0.017 (3)	0.005 (3)	0.025 (3)
C13	0.042 (7)	0.111 (14)	0.087 (7)	−0.004 (8)	−0.006 (5)	0.024 (10)
C14	0.111 (12)	0.102 (15)	0.104 (8)	0.051 (13)	−0.020 (8)	−0.006 (14)
C15	0.038 (3)	0.039 (3)	0.057 (3)	0.009 (2)	0.010 (2)	0.006 (3)
C16	0.052 (4)	0.046 (4)	0.059 (3)	0.013 (3)	0.003 (3)	0.010 (3)
C17	0.100 (6)	0.053 (5)	0.173 (8)	0.035 (5)	0.001 (6)	0.015 (5)
C18	0.119 (8)	0.082 (7)	0.259 (13)	0.024 (6)	−0.031 (8)	−0.033 (8)
C19	0.041 (3)	0.043 (3)	0.062 (3)	0.000 (3)	0.005 (3)	−0.006 (3)
C20	0.043 (3)	0.050 (4)	0.059 (3)	0.005 (3)	0.008 (3)	0.011 (3)
C21	0.048 (3)	0.048 (4)	0.046 (3)	0.007 (3)	0.005 (2)	0.002 (3)
C22	0.047 (3)	0.045 (3)	0.048 (3)	0.004 (3)	0.008 (3)	−0.005 (3)
C23	0.048 (4)	0.062 (4)	0.053 (3)	−0.004 (3)	0.000 (3)	−0.015 (3)
C24	0.059 (4)	0.086 (5)	0.045 (3)	0.012 (4)	−0.004 (3)	0.000 (3)
C25	0.068 (4)	0.062 (4)	0.054 (3)	0.010 (3)	−0.003 (3)	0.007 (3)
C26	0.051 (4)	0.059 (4)	0.052 (3)	0.001 (3)	0.004 (3)	0.003 (3)
C13'	0.042 (7)	0.111 (14)	0.087 (7)	−0.004 (8)	−0.006 (5)	0.024 (10)
C14'	0.111 (12)	0.102 (15)	0.104 (8)	0.051 (13)	−0.020 (8)	−0.006 (14)
N1	0.042 (3)	0.042 (3)	0.048 (3)	−0.009 (2)	0.0029 (19)	0.002 (2)
N2	0.035 (3)	0.040 (3)	0.056 (3)	0.000 (2)	0.010 (2)	0.006 (2)
N3	0.042 (3)	0.039 (3)	0.049 (2)	0.000 (2)	0.0075 (19)	0.006 (2)
N4	0.034 (2)	0.043 (3)	0.053 (3)	−0.001 (2)	0.004 (2)	0.002 (2)
O1	0.073 (3)	0.045 (3)	0.067 (2)	−0.016 (2)	0.001 (2)	0.009 (2)
O2	0.036 (2)	0.048 (2)	0.076 (2)	−0.0035 (17)	0.0042 (18)	−0.0036 (18)
O3	0.066 (3)	0.193 (6)	0.132 (4)	0.024 (3)	0.012 (3)	0.115 (4)
O4	0.036 (3)	0.176 (5)	0.076 (3)	0.017 (3)	−0.003 (2)	0.049 (3)
O5	0.079 (3)	0.046 (3)	0.152 (4)	0.023 (2)	0.031 (3)	0.031 (3)
O6	0.052 (3)	0.073 (3)	0.083 (3)	0.019 (2)	0.015 (2)	0.007 (2)

### Geometric parameters (Å, °)

**Table:**

Br1—C2	1.886 (6)	C14—H14B	0.9600
Br2—C5	1.899 (6)	C14—H14C	0.9600
Br3—C23	1.895 (6)	C15—N4	1.432 (6)
Br4—C26	1.912 (6)	C15—N3	1.442 (6)
C1—C2	1.391 (6)	C15—C16	1.540 (7)
C1—C6	1.415 (7)	C16—O6	1.183 (6)
C1—C8	1.513 (7)	C16—O5	1.320 (7)
C2—C3	1.394 (8)	C17—C18	1.374 (11)
C3—C4	1.352 (8)	C17—O5	1.466 (7)
C3—H3	0.9300	C17—H17A	0.9700
C4—C5	1.373 (8)	C17—H17B	0.9700
C4—H4	0.9300	C18—H18A	0.9600
C5—C6	1.382 (7)	C18—H18B	0.9600
C6—C7	1.504 (7)	C18—H18C	0.9600
C7—N1	1.458 (6)	C19—N4	1.444 (6)
C7—H7A	0.9700	C19—C22	1.522 (7)
C7—H7B	0.9700	C19—H19A	0.9700
C8—N2	1.464 (6)	C19—H19B	0.9700
C8—H8A	0.9700	C20—N3	1.449 (6)
C8—H8B	0.9700	C20—C21	1.516 (7)
C9—O1	1.205 (6)	C20—H20A	0.9700
C9—N1	1.370 (6)	C20—H20B	0.9700
C9—N3	1.379 (6)	C21—C26	1.386 (7)
C10—O2	1.213 (5)	C21—C22	1.399 (7)
C10—N4	1.371 (6)	C22—C23	1.380 (7)
C10—N2	1.376 (6)	C23—C24	1.387 (8)
C11—N1	1.437 (6)	C24—C25	1.365 (8)
C11—N2	1.441 (6)	C24—H24	0.9300
C11—C12	1.550 (7)	C25—C26	1.386 (7)
C11—C15	1.571 (7)	C25—H25	0.9300
C12—O3	1.178 (6)	C13'—O4	1.461 (8)
C12—O4	1.289 (6)	C13'—C14'	1.498 (9)
C13—O4	1.466 (8)	C13'—H13C	0.9700
C13—C14	1.496 (9)	C13'—H13D	0.9700
C13—H13A	0.9700	C14'—H14D	0.9600
C13—H13B	0.9700	C14'—H14E	0.9600
C14—H14A	0.9600	C14'—H14F	0.9600
			
Br4···O6^i^	3.349 (6)		
			
C2—C1—C6	119.4 (5)	H17A—C17—H17B	107.8
C2—C1—C8	121.2 (5)	C17—C18—H18A	109.5
C6—C1—C8	119.3 (5)	C17—C18—H18B	109.5
C1—C2—C3	120.6 (5)	H18A—C18—H18B	109.5
C1—C2—Br1	122.3 (4)	C17—C18—H18C	109.5
C3—C2—Br1	117.0 (4)	H18A—C18—H18C	109.5
C4—C3—C2	119.6 (6)	H18B—C18—H18C	109.5
C4—C3—H3	120.2	N4—C19—C22	114.8 (4)
C2—C3—H3	120.2	N4—C19—H19A	108.6
C3—C4—C5	120.5 (6)	C22—C19—H19A	108.6
C3—C4—H4	119.8	N4—C19—H19B	108.6
C5—C4—H4	119.8	C22—C19—H19B	108.6
C4—C5—C6	122.3 (6)	H19A—C19—H19B	107.6
C4—C5—Br2	116.3 (5)	N3—C20—C21	115.7 (4)
C6—C5—Br2	121.3 (5)	N3—C20—H20A	108.3
C5—C6—C1	117.5 (5)	C21—C20—H20A	108.3
C5—C6—C7	122.1 (5)	N3—C20—H20B	108.3
C1—C6—C7	120.4 (5)	C21—C20—H20B	108.3
N1—C7—C6	114.1 (4)	H20A—C20—H20B	107.4
N1—C7—H7A	108.7	C26—C21—C22	118.1 (5)
C6—C7—H7A	108.7	C26—C21—C20	121.5 (5)
N1—C7—H7B	108.7	C22—C21—C20	120.4 (5)
C6—C7—H7B	108.7	C23—C22—C21	119.6 (5)
H7A—C7—H7B	107.6	C23—C22—C19	120.8 (5)
N2—C8—C1	114.2 (4)	C21—C22—C19	119.6 (5)
N2—C8—H8A	108.7	C22—C23—C24	121.0 (5)
C1—C8—H8A	108.7	C22—C23—Br3	122.6 (5)
N2—C8—H8B	108.7	C24—C23—Br3	116.4 (4)
C1—C8—H8B	108.7	C25—C24—C23	120.1 (5)
H8A—C8—H8B	107.6	C25—C24—H24	120.0
O1—C9—N1	126.2 (5)	C23—C24—H24	120.0
O1—C9—N3	126.0 (5)	C24—C25—C26	119.1 (6)
N1—C9—N3	107.8 (5)	C24—C25—H25	120.5
O2—C10—N4	126.5 (5)	C26—C25—H25	120.5
O2—C10—N2	125.5 (5)	C25—C26—C21	122.1 (5)
N4—C10—N2	108.0 (4)	C25—C26—Br4	116.0 (4)
N1—C11—N2	114.3 (4)	C21—C26—Br4	121.9 (4)
N1—C11—C12	109.5 (4)	O4—C13'—C14'	99.8 (13)
N2—C11—C12	111.0 (4)	O4—C13'—H13C	111.8
N1—C11—C15	102.6 (4)	C14'—C13'—H13C	111.8
N2—C11—C15	103.6 (4)	O4—C13'—H13D	111.8
C12—C11—C15	115.6 (4)	C14'—C13'—H13D	111.8
O3—C12—O4	124.5 (5)	H13C—C13'—H13D	109.5
O3—C12—C11	123.8 (6)	C13'—C14'—H14D	109.5
O4—C12—C11	111.7 (5)	C13'—C14'—H14E	109.5
O4—C13—C14	101.1 (12)	H14D—C14'—H14E	109.5
O4—C13—H13A	111.6	C13'—C14'—H14F	109.5
C14—C13—H13A	111.5	H14D—C14'—H14F	109.5
O4—C13—H13B	111.6	H14E—C14'—H14F	109.5
C14—C13—H13B	111.6	C9—N1—C11	113.2 (4)
H13A—C13—H13B	109.4	C9—N1—C7	122.9 (4)
N4—C15—N3	114.0 (4)	C11—N1—C7	122.1 (4)
N4—C15—C16	111.8 (4)	C10—N2—C11	111.0 (4)
N3—C15—C16	110.9 (4)	C10—N2—C8	119.6 (4)
N4—C15—C11	102.3 (4)	C11—N2—C8	122.1 (4)
N3—C15—C11	103.2 (4)	C9—N3—C15	111.7 (4)
C16—C15—C11	114.2 (4)	C9—N3—C20	120.9 (4)
O6—C16—O5	126.0 (5)	C15—N3—C20	120.8 (4)
O6—C16—C15	125.0 (5)	C10—N4—C15	113.1 (4)
O5—C16—C15	109.0 (5)	C10—N4—C19	122.6 (4)
C18—C17—O5	112.9 (7)	C15—N4—C19	122.8 (4)
C18—C17—H17A	109.0	C12—O4—C13'	108.5 (8)
O5—C17—H17A	109.0	C12—O4—C13	122.8 (8)
C18—C17—H17B	109.0	C13'—O4—C13	34.4 (8)
O5—C17—H17B	109.0	C16—O5—C17	117.9 (5)
			
C6—C1—C2—C3	−2.1 (7)	C22—C21—C26—Br4	176.0 (4)
C8—C1—C2—C3	−178.9 (5)	C20—C21—C26—Br4	−2.7 (7)
C6—C1—C2—Br1	178.5 (3)	O1—C9—N1—C11	−175.0 (5)
C8—C1—C2—Br1	1.7 (6)	N3—C9—N1—C11	7.5 (5)
C1—C2—C3—C4	3.0 (8)	O1—C9—N1—C7	−10.2 (8)
Br1—C2—C3—C4	−177.6 (4)	N3—C9—N1—C7	172.3 (4)
C2—C3—C4—C5	−2.1 (9)	N2—C11—N1—C9	111.4 (4)
C3—C4—C5—C6	0.3 (8)	C12—C11—N1—C9	−123.3 (5)
C3—C4—C5—Br2	176.6 (4)	C15—C11—N1—C9	0.0 (5)
C4—C5—C6—C1	0.6 (7)	N2—C11—N1—C7	−53.6 (6)
Br2—C5—C6—C1	−175.5 (3)	C12—C11—N1—C7	71.8 (6)
C4—C5—C6—C7	178.7 (5)	C15—C11—N1—C7	−165.0 (4)
Br2—C5—C6—C7	2.6 (7)	C6—C7—N1—C9	−86.1 (6)
C2—C1—C6—C5	0.3 (7)	C6—C7—N1—C11	77.4 (6)
C8—C1—C6—C5	177.2 (4)	O2—C10—N2—C11	166.6 (5)
C2—C1—C6—C7	−177.9 (4)	N4—C10—N2—C11	−14.4 (5)
C8—C1—C6—C7	−0.9 (7)	O2—C10—N2—C8	15.8 (7)
C5—C6—C7—N1	120.7 (5)	N4—C10—N2—C8	−165.2 (4)
C1—C6—C7—N1	−61.2 (6)	N1—C11—N2—C10	−97.3 (5)
C2—C1—C8—N2	−120.9 (5)	C12—C11—N2—C10	138.3 (5)
C6—C1—C8—N2	62.2 (6)	C15—C11—N2—C10	13.6 (5)
N1—C11—C12—O3	−122.6 (7)	N1—C11—N2—C8	52.7 (6)
N2—C11—C12—O3	4.5 (9)	C12—C11—N2—C8	−71.8 (6)
C15—C11—C12—O3	122.1 (7)	C15—C11—N2—C8	163.5 (4)
N1—C11—C12—O4	56.7 (6)	C1—C8—N2—C10	71.0 (6)
N2—C11—C12—O4	−176.1 (5)	C1—C8—N2—C11	−76.5 (6)
C15—C11—C12—O4	−58.6 (7)	O1—C9—N3—C15	169.9 (5)
N1—C11—C15—N4	111.6 (4)	N1—C9—N3—C15	−12.6 (5)
N2—C11—C15—N4	−7.6 (5)	O1—C9—N3—C20	18.3 (7)
C12—C11—C15—N4	−129.3 (5)	N1—C9—N3—C20	−164.2 (4)
N1—C11—C15—N3	−7.0 (4)	N4—C15—N3—C9	−98.0 (5)
N2—C11—C15—N3	−126.3 (4)	C16—C15—N3—C9	134.8 (4)
C12—C11—C15—N3	112.1 (5)	C11—C15—N3—C9	12.1 (5)
N1—C11—C15—C16	−127.5 (5)	N4—C15—N3—C20	53.5 (6)
N2—C11—C15—C16	113.3 (5)	C16—C15—N3—C20	−73.6 (5)
C12—C11—C15—C16	−8.3 (7)	C11—C15—N3—C20	163.7 (4)
N4—C15—C16—O6	−129.1 (6)	C21—C20—N3—C9	73.2 (6)
N3—C15—C16—O6	−0.7 (8)	C21—C20—N3—C15	−75.8 (6)
C11—C15—C16—O6	115.4 (6)	O2—C10—N4—C15	−172.1 (5)
N4—C15—C16—O5	50.9 (6)	N2—C10—N4—C15	9.0 (5)
N3—C15—C16—O5	179.3 (4)	O2—C10—N4—C19	−5.5 (8)
C11—C15—C16—O5	−64.7 (6)	N2—C10—N4—C19	175.5 (4)
N3—C20—C21—C26	−118.9 (5)	N3—C15—N4—C10	110.2 (5)
N3—C20—C21—C22	62.5 (6)	C16—C15—N4—C10	−123.1 (5)
C26—C21—C22—C23	−1.8 (7)	C11—C15—N4—C10	−0.5 (5)
C20—C21—C22—C23	176.9 (5)	N3—C15—N4—C19	−56.3 (6)
C26—C21—C22—C19	178.8 (4)	C16—C15—N4—C19	70.4 (6)
C20—C21—C22—C19	−2.6 (7)	C11—C15—N4—C19	−167.0 (4)
N4—C19—C22—C23	122.6 (5)	C22—C19—N4—C10	−87.6 (6)
N4—C19—C22—C21	−58.0 (6)	C22—C19—N4—C15	77.6 (6)
C21—C22—C23—C24	3.9 (8)	O3—C12—O4—C13'	−17.0 (15)
C19—C22—C23—C24	−176.7 (5)	C11—C12—O4—C13'	163.7 (12)
C21—C22—C23—Br3	−175.9 (4)	O3—C12—O4—C13	18.0 (13)
C19—C22—C23—Br3	3.5 (7)	C11—C12—O4—C13	−161.4 (8)
C22—C23—C24—C25	−2.9 (9)	C14'—C13'—O4—C12	171.1 (16)
Br3—C23—C24—C25	176.9 (4)	C14'—C13'—O4—C13	49.6 (18)
C23—C24—C25—C26	−0.2 (9)	C14—C13—O4—C12	−116.9 (15)
C24—C25—C26—C21	2.4 (9)	C14—C13—O4—C13'	−43 (2)
C24—C25—C26—Br4	−175.1 (4)	O6—C16—O5—C17	−6.4 (10)
C22—C21—C26—C25	−1.4 (8)	C15—C16—O5—C17	173.6 (6)
C20—C21—C26—C25	180.0 (5)	C18—C17—O5—C16	102.1 (9)

Symmetry codes: (i) −*x*, −*y*, −*z*+2.

### Hydrogen-bond geometry (Å, °)

**Table:**

*D*—H···*A*	*D*—H	H···*A*	*D*···*A*	*D*—H···*A*
C4—H4···O2^ii^	0.93	2.36	3.278 (7)	172
C19—H19A···O3^iii^	0.97	2.27	3.227 (7)	169
C25—H25···O2^iv^	0.93	2.56	3.309 (7)	138

Symmetry codes: (ii) −*x*+1, *y*−1/2, −*z*+3/2; (iii) *x*, −*y*+1/2, *z*+1/2; (iv) −*x*+1, −*y*, −*z*+2.
